# Acknowledgement of Reviewers

**Published:** 2026-01-16

Dear Readers,

Our reviewers perform a very important and precious role in the evaluation of scientific articles and make valuable contributions to the increasing quality. Editorial Board would like to thank all the reviewers listed below for their support in Archives of Rheumatology in 2025

Abdulvahap Kahveci

Abdurrahman Tufan

Ahmet Cemal Pazarlı

Ahmet Kıvanç Menekşeoğlu

Ahmet Özgül

Akın Erdal

Alev Alp

Ali Karakaş

Ali S M Jawad

Alper Doğancı

Amra Adrovic

Andrea Tesija Kuna

Annasari Mustafa

Antoni Chan

Aslinur Keles

Ayhan Kamanlı

Aylin Rezvani

Aylin Sarıyıldız

Ayşe Merve Ata

Ayşe Ünal Enginar

Banu Kuran

Banu Ordahan

Berrin Yüksel

Bledjan Çuni

Bora Uzuner

Burak Okyar

Büşra Varman

Canan Şanal Toprak

Cem Zafer Yıldır

Charalampos Papagoras

Coşkun Zateri

David Kiefer

Dayawa D. Agoons

Dilek Eker Buyuksireci

Diogo Homann

Doo-ho Lim

Dragana Lazarevic

Durga Prasanna Misra

Duygu Ersözlü

Duygu Şilte Karamanlıoğlu

Ebru Atalar

Ece Cinar

Ece Kaptanoğlu

Eda Diyarbakır

Eduardo Collantes-estevez

Efe Sezgin

Elif Becenen Durmuş

Elif Tarihçi Çakmak

Elisa Belluzzi

Emine Uslu

Emrah Koç

Emre Bilgin

Ender Salbaş

Erhan Eser

Erkan Kılıç

Ernest H. Choy

Ezgi Agadayı

Ezgi Akyıldız Tezcan

Faisal Parlindungan

Fatih Baygutalp

Fatih Çay

Fatih Yıldız

Fatma Gül Yurdakul

Felipe Silva

Gamze Alaylı

Gamze Kılıç

Gizem Cengiz

Gökhan Sargın

Gülcan Gürer

Günay Yolcu

Gürkan Akgöl

Hakan Apaydın

Hakan Beyaztaş

Hatice Bodur

Hilal Ecesoy

Ilke Coskun Benlidayi

İsmail Sarı

İsmihan Sunar

İlhan Sezer

İlker İlhanlı

İlker Yağcı

Kazım Çapacı

Kemal Erol

Kemal Nas

Kevser Orhan

Kezban Armağan

Kıvanç Cengiz

Levent Karataş

Luis Catoggio

M. Erkan Kozanoğlu

Mahir Topaloğlu

Mahmoud A. Senousy

Maria Cristina Morán-Moguel

Mehmet Ali Balci

Mehmet Çağlayan

Mehmet Serkan Kılıçoğlu

Mehmet Yildiz

Meltem Karacaatlı

Meral Filiz

Mert Zure

Merve Damla Korkmaz

Mete Pekdiker

Metin Özgen

Mohamed Hussein

Mohammad Saeed

Mohammed Hassan Abu-Zaid

Mohammed Kamel

Mohd Shahrir Mohamed Said

Muhammed Mubarak

Murat Toprak

Musa Polat

Mustafa Dinler

Mustafa Turgut Yıldızgören

Müçteba Enes Yayla

Neslihan Gokcen

Nesrin Şen

Nihan Balta

Nihan Cuzdan Balta

Nuran Oz

Nurdan Kotevoğlu

Okan Kucukakkas

Özge Başaran

Özge Gülsüm İlleez

Özge Keniş Coşkun

Özgür Akgül

Özgür Kasapçopur

Özlem Kılıç

Özlem Özdemir Işık

Rabia Miray Kisla Ekinci

Rabia Sanır

Remzi Çevik

Rengin Güzel

Reyhan Dedeoğlu

Reyhan Kose

Rezan Koçak Ulucaköy

Rıza Can Kardaş

Roberto Giacomelli

Sadaf Alipour

Safaa Mahran

Salih Özgöçmen

Sanem Aykan

Seçilay Güneş

Selçuk Yüksel

Selda Çiftci İnceoğlu

Selim Nalbant

Sema Koc Yildirim

Senem Sas

Serkan Küççüktürk

Sertaç Ketenci̇

Servet Yolbas

Sevilay Batıbay

Sezgin Şahin

Sezgin Zontul

Sham Santhanam

Sibel Bakırcı

Sibel Doğan

Süleyman Gül

Tatjana Zekic

Tuba Guler

Tugba Izci Duran

Tugba Ozsoy- Unubol

Tuğba Atan

Umut Bakay

Ülkü Uçar

Veysel Alcan

Worawit Louthrenoo

Yasemin Ulus

Zafer Günendi

Zerrin Kasap

Zülfinaz Betul Celik

